# Warburg effect in chemosensitivity: Targeting lactate dehydrogenase-A re-sensitizes Taxol-resistant cancer cells to Taxol

**DOI:** 10.1186/1476-4598-9-33

**Published:** 2010-02-09

**Authors:** Ming Zhou, Yuhua Zhao, Yan Ding, Hao Liu, Zixing Liu, Oystein Fodstad, Adam I Riker, Sushama Kamarajugadda, Jianrong Lu, Laurie B Owen, Susan P Ledoux, Ming Tan

**Affiliations:** 1 Mitchell Cancer Institute, University of South Alabama, Mobile, Alabama, USA; 2 Department of Cell Biology and Neuroscience, University of South Alabama, Mobile, Alabama, USA; 3 Cancer Research Institute, Central South University, Changsha, China; 4 Institute for Cancer Research, The Norwegian Radium Hospital, University of Oslo, Norway; 5 Department of Biochemistry and Molecular Biology, University of Florida, Gainesville, Florida, USA; 6 Ochsner Cancer Institute, Ochsner Health System, New Orleans, Louisiana, USA

## Abstract

**Background:**

Taxol is one of the most effective chemotherapeutic agents for the treatment of patients with breast cancer. Despite impressive clinical responses initially, the majority of patients eventually develop resistance to Taxol. Lactate dehydrogenase-A (LDH-A) is one of the predominant isoforms of LDH expressed in breast tissue, which controls the conversion of pyruvate to lactate and plays an important role in glucose metabolism. In this study we investigated the role of LDH-A in mediating Taxol resistance in human breast cancer cells.

**Results:**

Taxol-resistant subclones, derived from the cancer cell line MDA-MB-435, sustained continuous growth in high concentrations of Taxol while the Taxol-sensitive cells could not. The increased expression and activity of LDH-A were detected in Taxol-resistant cells when compared with their parental cells. The downregulation of LDH-A by siRNA significantly increased the sensitivity of Taxol-resistant cells to Taxol. A higher sensitivity to the specific LDH inhibitor, oxamate, was found in the Taxol-resistant cells. Furthermore, treating cells with the combination of Taxol and oxamate showed a synergistical inhibitory effect on Taxol-resistant breast cancer cells by promoting apoptosis in these cells.

**Conclusion:**

LDH-A plays an important role in Taxol resistance and inhibition of LDH-A re-sensitizes Taxol-resistant cells to Taxol. This supports that Warburg effect is a property of Taxol resistant cancer cells and may play an important role in the development of Taxol resistance. To our knowledge, this is the first report showing that the increased expression of LDH-A plays an important role in Taxol resistance of human breast cancer cells. This study provides valuable information for the future development and use of targeted therapies, such as oxamate, for the treatment of patients with Taxol-resistant breast cancer.

## Background

Taxol (paclitaxel) has recently emerged as an important agent in the treatment of human breast cancer as well as other tumor histologies, such as ovarian, prostate and non-small cell lung cancers [1,2]. The primary cellular targets of Taxol are the microtubules of cancer cells, which is vital for mitotic activity, cellular motility and proliferative capacity. Taxol stabilizes the microtubule structure by disrupting the dynamic equilibrium between soluble tubulin dimers and their polymerized form. It is also a potent inhibitor of chromosomal replication by blocking cells in the late G2 or mitotic phases of the cell cycle [3]. The resistance of cancer cells to Taxol and other chemotherapeutic agents is known to result in the subsequent recurrence and metastasis of cancer [4,5]. One known mechanism involved with cancer cell resistance to Taxol and other microtubule-stabilizing agents is the high-expression of the membrane P-glycoprotein that functions as a drug-efflux pump [6]. Other cellular mechanisms include the alterations of tubulin structure [7-9], changes in the drug-binding affinity of the microtubules [10] and cell cycle deregulation [11,12]. However, the detailed molecular mechanisms that may contribute to Taxol resistance of cancer cells are still not fully understood.

Cancer cells, unlike their normal counterparts, use aerobic glycolysis with reduced mitochondrial oxidative phosphorylation for glucose metabolism. This persistence of high lactate production by cancer cells in the presence of oxygen, known as aerobic glycolysis, was first noted by Otto Warburg more than 75 years ago [13-15]. It was recognized that since cancer cells have increased cell growth and energy needs to sustain cell proliferation, elevated glycolytic activity insures that adequate ATP levels are available to meet the demands of rapidly proliferating tumor cells within a hypoxic microenvironment [16]. Additionally, Taxol-resistant cancer cells may escape the therapeutic effects of Taxol via the efflux transport systems present within tumor cells. However, drug efflux and metabolism consumes large amounts of ATP that is generated via glycolysis, protecting cells from the lethal effects of Taxol by sustaining the energy needed for cellular drug efflux and metabolism. Thus, the energy distribution consumed in Taxol-resistant cells must be dramatically altered in order to accommodate for both cell viability and long-term survival.

Lactate dehydrogenase-A (LDH-A) is one of the main isoforms of LDH expressed in breast tissue, controlling the conversion of pyruvate to lactate of the cellular glycolytic process [17]. It has been shown that LDH-A plays a key role in glycolysis, growth properties and tumor maintenance of breast cancer cells [16,18]. To understand the cellular mechanisms involved in the resistance of breast cancer cells to Taxol, we investigated on the association of LDH-A and Taxol resistance in breast cancer cells and the role of LDH-A in tumor therapeutics and drug sensitivity. Our results show that compared with their parental cells, the increased expression and activity of LDH-A in Taxol-resistant cells directly correlate with their sensitivity to glycolysis inhibitor oxamate. Furthermore, gene expression knockdown experiments with siRNA specific for LDH-A show an increased sensitivity of these cells to Taxol. In addition, treatment of breast cancer cells with the combination of Taxol with oxamate, reveals an synergistically inhibitory effect upon cell viability. Taken together, LDH-A plays an important role in Taxol resistance of breast cancer cells, serving as a promising therapeutic target for overcoming Taxol resistance. Furthermore, the data are consistent with the role of LDH-A as an essential tumor maintenance gene, providing further insight into the cellular and molecular mechanisms involved in Taxol-resistant breast cancer.

## Methods

### Cells and cell culture

Breast cancer cells MDA-MB-435 (MDA-435), MDA-MB-231 (MDA-231), MCF7 and BT474 were purchased from American Type Culture Collection (ATCC). 435TR1 and 435TRP cells are Taxol-resistant single clone or pooled clones, which were developed from parental MDA-435 cells by treated with gradually increasing concentrations of Taxol in cell culture medium. MDA-231 cell line with stable knockdown of LDH-A was constructed through transfection of MSCV-based retroviral vector (MSCV/LTRmiR30-PIG). All of these cells were cultured in DMEM/F-12 (Mediatech Inc.) and supplemented with 10% FBS and Penicillin/Streptomycin.

### Morphological observation of Taxol-resistant cells

The cells were seeded in 6-well plates at 3 × 10^5 ^cells per well in duplicate. After 12 hr incubation, cells were treated with or without 20 nM Taxol for 24 hrs, with untreated cells serving as controls. The cells were washed twice with PBS and then fixed with methanol/acetone (1:1), subsequently stained with 4',6-diamidino-2-phenylindole (DAPI) in order to visualize the morphology of cell nucleus. The morphology of cells was observed with the fluorescence microscope.

### Cell apoptosis assay

The cancer cells were treated with 20 nM Taxol for 48 hrs. Two methods were used to detect apoptosis. 1) The early stage of apoptosis was detected by Annexin V/propidium iodide staining with the Apoptosis Detection Kit (BD PharMingen). Briefly, aliquots of 10^5 ^Taxol-treated cells were incubated with Annexin V/propidium iodide for 15 min at room temperature. The cells were then analyzed by flow cytometry (BD LSR II). 2) The late stage of apoptosis was detected by Cell Death Detection ELISA PLUS kit (Roche) according to the manufacturer's instruction.

### Western blotting

Cells were harvested and lysed in a buffer containing 50 mM Tris-HCl, pH 7.5, 150 mM NaCl, 2 mM EDTA, 1% Triton, 1 mM PMSF and Protease Inhibitor Cocktail (Sigma) for 20 min on ice. Lysates were cleared by centrifugation at 14,000 rpm at 4°C for 10 min. Supernatants were collected and protein concentrations were determined by the Bradford assay (Bio-rad). The proteins were then separated with a SDS/polyacrylamide gel and transferred to a Nitrocellulose membrane (Bio-rad). After blocking in PBS with 5% non-fat dry milk for 1 hr, the membranes were incubated overnight at 4-8°C with the primary antibodies in PBS with 5% non-fat dry milk. The following antibodies were utilized: anti-LDHA rabbit antibody (1:1000, Cell Signaling); anti-PARP rabbit antibody (1:1000, Cell Signaling), anti-cleaved PARP Rabbit antibody (1:1000, Cell Signaling), anti-Bcl2 rabbit antibody (1:1000, Cell Signaling), anti-Bcl-XL rabbit monoclonal antibody (1:1000, Cell Signaling), anti-Cdc2 mouse monoclonal antibody (1:1000, Cell Signaling),, anti-p-Cdc2(Y15) rabbit monoclonal antibody (1:1000, Cell Signaling), and anti-β-actin monoclonal antibody (1:2000, Sigma). Membranes were extensively washed with PBS and incubated with horseradish peroxidase conjugated secondary anti-mouse antibody or anti-rabbit antibody (1:2,000, Bio-rad). After additional washes with PBS, antigen-antibody complexes were visualized with the enhanced chemiluminescence kit (Pierce).

### Detection of LDH Activity

The total LDH activity in cell lysates was examined according to the manufacturer's instructions of the LDH-cytotoxicity assay kit (BioVision). Briefly, 2 × 10^5 ^cells were seeded in a 24-well plate one day before assaying and all samples were analyzed in triplicate. Then cells were collected, washed and extracted for protein to measure LDH activity. Results were normalized based upon total protein.

### siRNA Experiments

siRNA oligonucleotides for LDH-A was purchased from Sigma, with a scrambled siRNA (Sigma) used as a control. Transfection was performed using the Oligofectamine Transfection reagent (Invitrogen) according to the manufacturer's protocol. Forty-eight hours after transfection, whole-cell lysates were prepared for further analysis by Western blot, LDH activity and Taxol cytotoxicity assay.

### Cell Viability Assay

A total of 5 × 10^3 ^~ 1 × 10^4 ^cells/well were seeded in 96-well plates. Twenty-four hours later, the medium was replaced with fresh medium with or without Taxol and incubated for 24 or 48 hrs, respectively. Taxol in combination with various concentrations of oxamate were also used to treat the cells in order to investigate the effect of drug combinations. Cell viability was determined by two methods. 1) Using CellTiter 96 Aqueous One Solution Cell Proliferation Assay (Promega) according to the manufacturer's protocol; 2) by Typan Blue staining and direct cell counting using hematocytometer.

### Statistical analysis

The unpaired Student's *t*-test was used for the data analysis. All data were shown as mean ± standard error (SE). A statistical difference of *P *< 0.05 was considered significant.

## Results

### Selection and characterization of Taxol-resistant cancer cells

MDA-435 cells were treated with gradually increasing concentrations of Taxol in cell culture medium for selection of Taxol-resistant cells. After successive Taxol treatments for duration of 3 months, several resistant cell clones were developed from the MDA-435 cell line. Taxol-resistant clone 1 (435TR1) and Taxol-resistant pooled clones (435TRP) were used for all subsequent experiments in this study.

**Figure 1:**
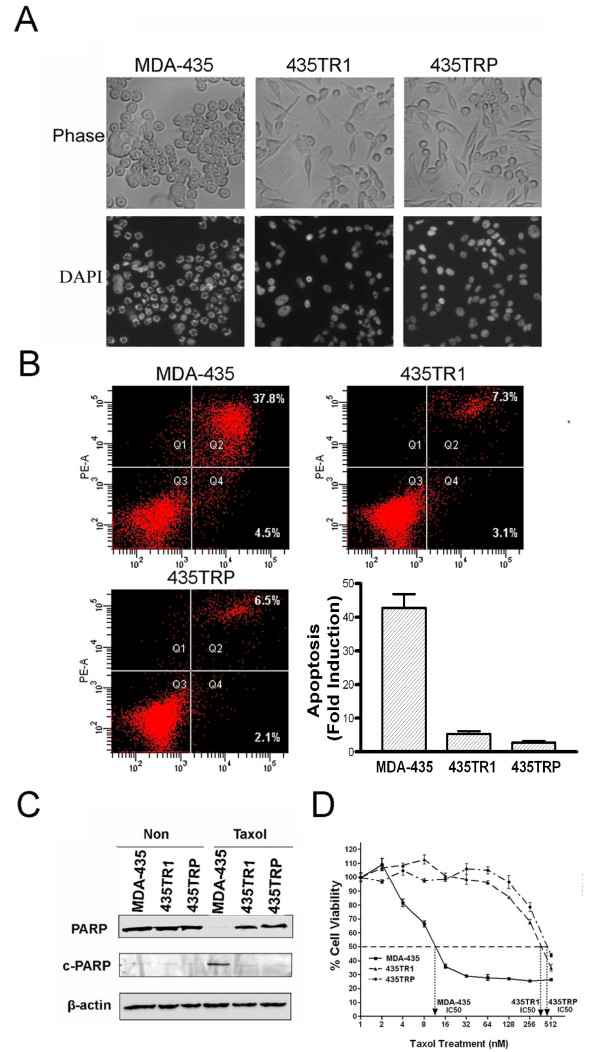
**Characterization of Taxol-resistant cells**. A, MDA-435, 435TR1 and 435TRP cells were treated with 20 nM Taxol for 24 hrs and their morphology was observed under fluorescence microscope. The phase image of these cells was shown at the top and the nucleus stained by DAPI was shown at the bottom (200 ×). B, MDA-435, 435TR1 and 435TRP cells were treated with 20 nM Taxol for 48 hrs and apoptosis was examined by flow cytometry using Annexin V/PI staining and by Cell Death Detection ELISA PLUS Kit. Fold induction value was calculated following the formula: mU of the sample (cells treated with Taxol)/mU of the corresponding negative control (cells without Taxol treatment). C, Taxol-resistant cells and their parental cells were treated without or with 20 nM Taxol for 48 hrs, then poly (ADP-ribose) polymerase (PARP) and its cleaved protein (c-PARP) were analyzed by Western blotting with specific antibodies, respectively. β-actin was used as a loading control. D, Cell viability analysis was performed to evaluate cytotoxicity of Taxol to MDA-435 and Taxol-resistant 435TR1 and 435TRP cells under treatment with indicated concentrations of Taxol for 48 hrs.

To compare the survival capacity of both Taxol-sensitive and Taxol-resistant cells, MDA-435, 435TR1 and 435TRP cells were treated with 20 nM Taxol for 24 hrs. Taxol-sensitive MDA-435 cells showed cell rounding and blebbing with empty spaces visualized within the cells. This suggested that a large portion of these cells were arrested in G2/M phase, with some of these cells undergoing apoptosis. However, no obvious morphological change was observed in Taxol-resistant 435TR1 and 435TRP cells (Fig. 1A). Early stage apoptosis was examined by flow cytometry analysis after staining with Annexin V/propidium iodide, and late stage apoptosis was detected by a Cell Death Detection ELISA PLUS kit, which examines the DNA fragmentation in the apoptotic cells. Both assays detected a smaller percentage of apoptotic cells in Taxol-resistant 435TR1 and 435TRP, compared to their parental MDA-435 cells after treatment with 20 nM Taxol for 48 hrs (Fig. 1B). The protein expression of the cleaved Poly (ADP-ribose) polymerase (c-PARP), an important marker of caspase-mediated apoptosis [19,20], was also examined by Western blotting after the cells were treated with 20 nM Taxol for 48 hrs. We found much lower levels of cleaved PARP and correspondingly much higher levels of un-cleaved PARP in Taxol-resistant 435TR1 and 435TRP cells, compared to parental MDA-435 cells (Fig. 1C). Cell viability assay showed that 435TR1 and 435TRP cells could tolerate much higher concentrations of Taxol compared to MDA-435 cells, with their IC50 concentrations found to be more than 30-fold higher than those of MDA-435 cells (Fig. 1D).

### Increased expression and activity of LDH-A in Taxol-resistant cells

**Figure 2:**
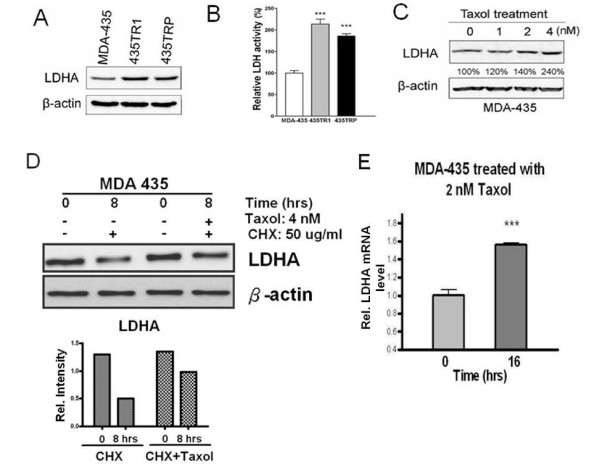
**Increased LDH-A expression and activity in Taxol-resistant cells**. A, Western blot was performed with an anti-LDH-A antibody of total cell extract from MDA-435, 435TR1 and 435TRP cells. The β-actin protein was used as a loading control. B, LDH activity in MDA-435, 435TR1 and 435TRP were examined. C, MDA-435 cells were treated with increasing concentrations of Taxol for 24 hrs. The cell lysates were prepared and Western blotting was carried out with antibodies against to LDH-A and β-actin. D, LDHA protein stability assay was performed in MDA-435 cells under the treatments of Taxol at 4 nM and CHX at 50 ug/ml followed by Western blotting assay to exam the protein expression level of LDHA at 0 and 8 hrs (top). The relative intensity of LDHA band was normalized to its β-actin loading (bottom). E, LDHA mRNA level was detected by real-time PCR under 2 nM Taxol in MDA-435 cells. The LDHA primers used for PCR are: forward, 5'-tgg agt gga atg aat gtt gc-3'; reverse: 5'-ata gcc cag gat gtg tag cc-3'. *Columns*, mean of three independent experiments; *bars*, SE. ***, *P *< 0.001.

To examine the role of LDH-A in mediating Taxol resistance in human breast cancer cells, the expression of LDH-A was examined in MDA-435, 435TR1 and 435TRP cells. We found that LDH-A levels were markedly increased in 435TR1 and 435TRP cells, compared to their parental MDA-435 cells (Fig. 2A). The activity of LDH was also increased about 2-fold in Taxol-resistant 435TR1 and 435TRP cells, compared to MDA-435 cells (Fig. 2B). These results indicated that Taxol resistance is correlated with the increased LDH-A expression and activity. Interestingly, treatment with Taxol resulted in the induction of LDH-A expression in a dose-dependent pattern in MDA-435 cells (Fig. 2C). We also identified that LDH activity could also be induced by Taxol in the Taxol-resistant cells (data not shown). To study the mechanism that may contribute to the increased expression and activity of LDH-A, MDA-435 cells were treated with CHX to block protein synthesis and the cells were further treated with or without Taxol for different times, the protein stability of LDH-A was measured by Western blot (Fig. 2D). The result showed that LDH-A protein is more stable in Taxol treated cells than that of untreated cells. We further compared the mRNA level of LDH-A in Taxol-treated and -untreated cells by qRT-PCR (Fig. 2E). The result showed that Taxol treatment increased the mRNA expression of LDH-A. These results suggest that both protein stability and mRNA induction by Taxol contribute to the up-regulation of LDH-A in these cells.

### The downregulation of LDH-A re-sensitizes Taxol-resistant cells to Taxol

**Figure 3:**
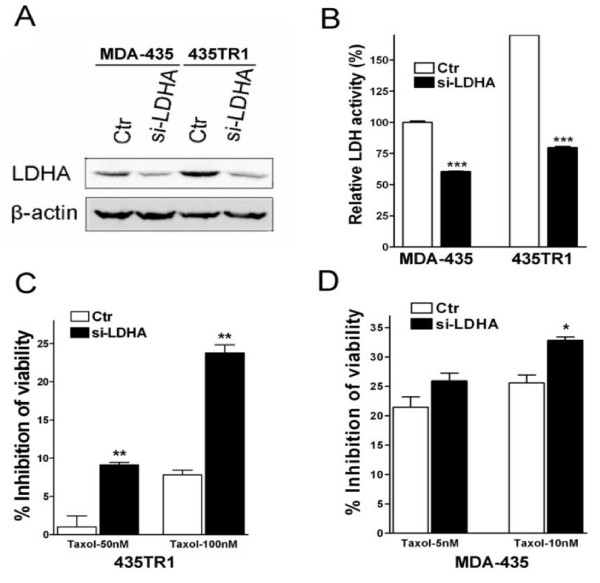
**Knockdown of LDH-A increases the sensitivity of Taxol-resistant 435TR1 cells to Taxol**. A, MDA-435 and 435TR1 cells were transfected with scramble siRNA (Ctr) or LDH-A siRNA. 48 hrs after siRNA transfection, cell lysates were prepared and Western blotting was performed with antibodies against LDH-A. The β-actin protein was used as a loading control. B, LDH activity was examined from lysates of MDA-435 and 435TR1 48 hrs after siRNA transfection. C and D, 24 hrs after siRNA transfection, MDA-435 and 435TR1 cells were seeded into 96-well plates at the density of 8 × 10^3 ^cells per well, and treated with Taxol (5 nM and 10 nM for MDA-435, 50 nM and 100 nM for 435TR1) for 48 hrs. Then the cell viability was detected using a MTS reagent. Data are presented as the percentage of viability inhibition measured in cells treated without Taxol. *Columns*, mean of three independent experiments; *bars*, SE. *, *P *< 0.05, **, *P *< 0.01, ***, *P *< 0.001.

**Figure 4:**
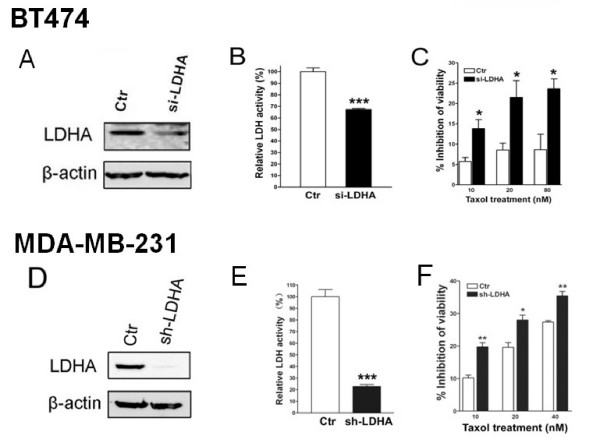
**Knockdown of LDH-A increases the sensitivity of breast cancer cell lines BT474 and MDA-231 to Taxol**. A, BT474 cells were transfected with scramble siRNA (Ctr) or si-LDHA. 48 hrs after siRNA transfection, cell lysates were prepared and immunoblot analyses were carried out with antibodies against LDHA and β-actin. B, 48 hrs after siRNA transfection, cell lysates were prepared and LDHA activity was examined. Data are shown in percentage of LDH activity relative to Ctr-transfected cells. C, 48 hrs after siRNA transfection, cells were seeded into 96-well plates at the density of 8 × 10^3 ^cells per well. 12 hrs after incubation, the cells were then treated with various concentrations of Taxol for 48 hrs. Then the cell viability was detected using a MTS reagent. Data are presented as the percentage of viability inhibition measured in cells treated without Taxol. D, LDHA protein expression in MDA-MB-231 (Ctr) and stable LDHA knowdown MDA-MB-231 cells (sh-LDHA) were detected to evaluate the efficiency of LDH-A knockdown by using an LDHA antibody. β-actin was used as a loading control. E, LDH activity was examined in MDA-MB-231 cells with and without stably knockdown of LDH-A. Data are shown in percentage of LDH activity relative to Ctr cells. F, MDA-MB-231 cells with or without stably knockdown of LDH-A were seeded into 96-well plates at the density of 8 × 10^3 ^cells per well. 12 hrs after incubation, the cells were treated with various concentrations of Taxol for 48 hrs. Then the cell viability was detected using a MTS reagent. Data are presented as the percentage of viability inhibition measured in cells treated without Taxol. *Columns*, mean of three independent experiments; *bars*, SE.*, *P *< 0.05, **, *P *< 0.01, ***, *P *< 0.001.

The increase of LDH-A expression and LDH activity detected in Taxol-resistant cells suggests that LDH-A may play a critical role in Taxol resistance. Therefore, the effect of LDH-A downregulation on the sensitivity of Taxol was investigated. After LDH-A was downregulated efficiently by specific siRNA to LDH-A (Fig. 3A), LDH activity was decreased about 40% in MDA-435 cells and about 55% in 435TR1 cells (Fig. 3B). Since the expression and activity of LDH-A was upregulated in Taxol-resistant cancer cells (Fig. 2), we hypothesized that the downregulation of LDH-A by siRNA might re-sensitize Taxol-resistant cells to Taxol. To this end, LDH-A was knocked down with siRNA in 435TR1 and parental MDA-435 cells respectively, and then the cells were treated with different concentrations of Taxol. The downregulation of LDH-A increased the sensitivity of these cells to Taxol, with Taxol-resistant 435TR1 cells showing about a 3-10 fold increase in cell growth inhibition under 50-100 nM Taxol treatment measured by both MTS assay (Fig. 3C) and direct cell counting (Additional file 1, Figure. S1). Interestingly, 435TR1 cells showed a much greater overall increased sensitivity to Taxol compared to their parental MDA-435 cells (Fig. 3C and 3D). Similar assays were performed in another breast cancer cell line BT474 (Fig. 4A-C), where the knockdown of LDH-A expression by siRNA increased the sensitivity to Taxol by at least 2-fold. To further confirm these results, MDA-231 cells with stable knockdown of LDH-A by short-hairpin RNA (shRNA) were used. Compared to those of control MDA-231 cells, LDH-A expression (Fig. 4D) and LDH activity (Fig. 4E) were dramatically decreased in LDH-A stably knockdown cells and these cells showed a much greater overall increased sensitivity to Taxol (Fig. 4F). These results demonstrated that LDH-A plays an important role in Taxol resistance. Since LDH is a critical enzyme in the glycolytic pathway, our results suggest that inhibition of glycolysis may re-sensitize Taxol-resistant cells to Taxol.

### The combination of Taxol with oxamate shows synergistic inhibitory effect on breast cancer cells

**Figure 5:**
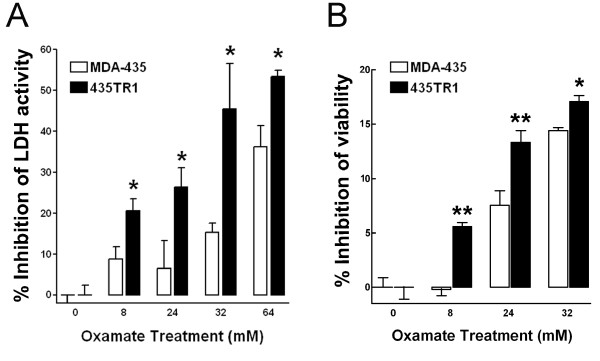
**Taxol-resistant cells are more sensitive to glycolysis inhibitor oxamate**. A, MDA-435 and 435TR1 cells were treated with various concentrations of oxamate for 48 hrs, then LDH activity was detected. Data are shown as the percentage of LDH activity inhibition detected in cells treated without oxamate. B, MDA-435 and 435TR1 were treated with various concentrations of oxamate for 48 hrs. Then the cell viability was detected using a MTS reagent. Data are presented as the percentage of viability inhibition measured in cells treated without oxamate. *Columns*, mean of three independent experiments; *bars*, SE. *, *P *< 0.05. **, *P *< 0.01.

Oxamate is a pyruvate analog that directly inhibits the converting process of pyruvate to lactate by LDH, therefore, inhibits cell glycolysis [21]. We first examined the effect of oxamate on LDH activity and cell viability of MDA-435 and 435TR1 cells. Oxamate treatment led to a decrease of LDH activity (Fig. 5A) and an inhibition of cell viability (Fig. 5B) in a dose-dependant manner, in both MDA-435 and 435TR1 cells. Compared to MDA-435 cells, Taxol resistant 435TR1 cells showed a greater sensitivity to oxamate, consistent with the results of LDH-A knockdown by siRNA (Fig. 3). Since glycolysis and mitochondrial oxidative phosphorylation are linked processes [16], and we have previously shown that LDH-A is critical in regulating glycolysis and growth of breast cancer cells [18], we reasoned that the increased expression and activity of LDH-A in Taxol-resistant cells may lead to an increase of glycolysis and a decrease of mitochondrial oxidative phosphorylation. Thus, a specific inhibitor of the mitochondrial oxidative phosphorylation, oligomycin was utilized to treat these cells. As expected, Taxol-resistant 435TR1 cells were more resistant to oligomycin (Additional file 2, Figure. S2). These results further support the notion that increased Taxol sensitivity by oxamate is a consequence of the inhibition of cellular glycolysis.

**Figure 6:**
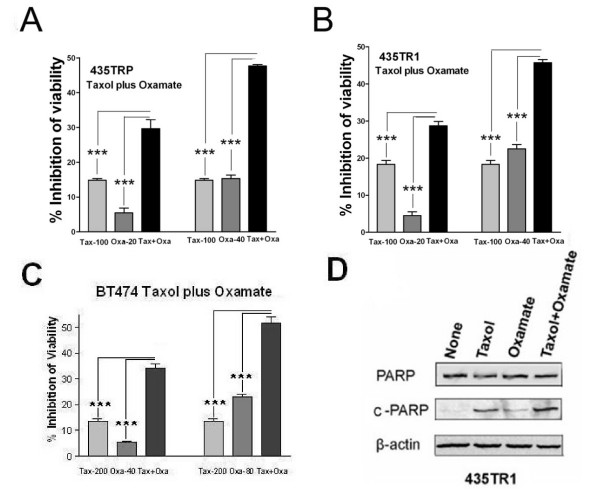
**Combination of Taxol with oxamate shows synergistic inhibitory effects of Taxol-resistant and BT474 cells**. A and B, 8 × 10^3 ^per well of 435TRP and 435TR1 cells were plated in 96-well plates and then treated with Taxol (Tax), Oxamate (Oxa) alone or Taxol plus Oxamate (Tax+ Oxa) with the indicated concentrations for 48 hrs. Cell viability was examined by MTS assay. Data are presented as the percentage of viability inhibition measured in cells treated without Tax and Oxa. C, BT474 cells were treated with Tax, Oxa alone or Tax plus Oxa with the indicated concentrations for 48 hrs.. Cell viability was measured by direct cell counting. Data are presented as the percentage of viability inhibition counted in cells treated without Tax and Oxa. D, 435TR1 cells were treated with 100 nM Taxol or/and 40 mM oxamate for 48 hrs, cell lysates were prepared and Western blotting were carried out with antibodies against total PARP (Top) or its cleaved protein c-PARP (Middle). β-actin was used as a loading control (Bottom). *Columns*, mean of three independent experiments; *bars*, SE. ***, *P *< 0.001.

Since downregulation of LDH-A by siRNA or oxamate significantly inhibited the viability of the Taxol-resistant cells, we further investigated the effects of combining Taxol with glycolysis inhibitor oxamate on Taxol-resistant breast cancer cells. In both Taxol-resistant 435TRP and 435TR1 cells (Fig. 6A and 6B; Additional file 3, Figure. S3), and in BT474 cells (Fig. 6C), Taxol combined with oxamate were much more effective in inhibiting cell viability compared with either agent given alone. Similar treatment combinations were performed in another breast cancer cell line, MCF7, with similar results obtained (Additional file 4, Figure. S4). Taken together, the combination of Taxol with oxamate has a greater capacity to inhibit Taxol-resistant cells compared to either agent given alone.

To further investigate the mechanism of oxamate-induced Taxol re-sensitization, we examined cellular apoptosis in these cells. PARP, a nuclear protein that can be easily cleaved by caspases, has been widely used as an apoptosis marker [19,20]. The expression level of total PARP and cleaved PARP (c-PARP) were examined in 435TR1 cells after treatment with Taxol, oxamate, or their combination for 48 hrs, respectively. We found a significant increase of the levels of cleaved PARP after treatment with the combination of Taxol and oxamate compared to treatment with single agent (Fig. 6D). This indicates that cellular apoptosis is a mechanism involved in the increased cell growth inhibitory effect of the combination treatment of Taxol with oxamate.

## Discussion

In this study, we investigated the role of LDH-A in the acquired Taxol resistance in multiple human breast cancer cell lines. We identified that compared to Taxol-sensitive cells, Taxol-resistant cells possess an increased expression and activity of LDH-A, with its downregulation resulting in an increased sensitivity of Taxol resistant-cells to Taxol. In addition, compared to Taxol-sensitive cells, Taxol-resistant cells show a higher sensitivity to the LDH inhibitor oxamate. Furthermore, when compared to single agent therapy, treating cells with the combination of Taxol and oxamate show a much stronger inhibitory effect on Taxol-resistant breast cancer cells by promoting cellular apoptosis. These results demonstrate that LDH-A plays an important role in Taxol resistance and potentially it can serve as a therapeutic target for overcoming Taxol resistance in patients with breast cancer.

Taxol is a widely used chemotherapeutic agent for the treatment of several types of cancers, including breast cancer. Taxol resistance may result in the subsequent recurrence and metastasis of cancer, ultimately resulting in death. Although extensive investigations have been done in regards to the resistance of cancer cells to Taxol, the specific mechanisms involved are still poorly understood. Cancer cells are different from non-neoplastic cells in their metabolic properties, with normal cells relying primarily on the process of mitochondrial oxidative phosphorylation, consuming oxygen and glucose to produce energy. In contrast, cancer cells depend mostly upon glycolysis, the anaerobic breakdown of glucose into the energy-storing molecule ATP, even in the presence of available oxygen [13-15,22,23]. Recently, research endeavors have been actively tried to make use of these unique bioenergetic properties to enhance the therapeutic efficacy of killing cancer cells.

LDH-A is one of the main isoforms of LDH expressed in breast tissue, catalyzing the conversion of pyruvate to lactate [17]. We and others have previously shown that LDH-A plays a critical role in glycolysis, growth properties and tumor maintenance of breast cancer cells [16,18]. Studies have shown that the LDH-A expression in cancer cells is associated with radiosensitivity [24]. LDH-A inhibition results in increased apoptosis via ROS production in cell with fumarate hydratase deficiency and was viewed as a therapeutic strategy for treatment of hereditary leiomyomatosis and renal cell cancer [25]. However, the role of LDH in Taxol resistance of cancer cell has not been explored. In this study, we selected a panel of Taxol-resistant cells by gradually increasing the concentration of Taxol in the cell culture medium. We used these, and other three breast cancer cell lines, to study the expression and activity of LDH-A in the development of Taxol resistance. To our knowledge, this is the first report to provide direct evidence in support of a role for LDH-A in acquired Taxol resistance in human breast cancer cells.

We found that Taxol treatment resulted in the increased LDH-A expression and activation in cancer cells, which appears as a result of the induction of LDH-A mRNA expression by Taxol. The downregulation of LDH-A by LDH-A siRNA and inhibition of LDH by oxamate led to increased sensitivity to Taxol in all three breast cancer cell lines examined in this study. This indicated that Taxol treatment triggers a feedforward cycle in which Taxol-induced activation of LDH results in cancer cells better survival under Taxol treatment, likely through promoting cell glycolysis. A recent study has shown that cancer cells inhibit cytochrome *c*-mediated apoptosis by a mechanism through deregulated glucose metabolism [26]. Thus, the Taxol-induced high expression and activity of LDH-A detected in Taxol-resistant cells could be a way of adaptation of these cells to Taxol treatment and to modulate glucose metabolism and glycolysis to avoid apoptosis induced by Taxol. Targeting LDH by LDH siRNA or LDH inhibitor oxamate interrupts the feedforward cycle and renders the re-sensitization to Taxol. These results indicate that LDH may potentially serve as an excellent target for overcoming Taxol resistance in human breast cancer patients.

Up-regulation of antiapoptotic Bcl-2 family members, such as Bcl-2 and Bcl-XL, was reported to contribute to Taxol-induced apoptosis [27]. In addition, we previously reported that the phosphorylation on tyrosine-15 of Cdc2 by ErbB2 in breast cancer cells resulting a delayed M phase entry and leading to an increased Taxol resistance [11]. We found that compared to the parental MDA435 cells, Taxol-resistant MDA435TR1 and MDA435TRP cells express lower Bcl-2 and lower phosphorylation level of Cdc2 at tyrosine-15 (Additional file 5, Figure. S5). Based on the known functions of Bcl2 and Y15-Cdc2 in Taxol resistance, these results can not explain the increased resistance in MDA435TR1 and MDA435TRP cells. However, we found that Bcl-XL was upregulated in Taxol-resistant cells (Additional file 5, Figure. S5). This might be another reason in addition to LDH-A for the increased Taxol resistance in these cells. It will be interesting to examine the relationship between LDH-A and Bcl-XL in these cells in our future studies.

The differences in cytotoxicity were some what modest when the LDH-A were knocked down by siRNA. One of the reasons might be the relatively low sensitivity of MTS assay to detect cell toxicity in our experiments. Another possible reason might be the relatively low knocking down efficiency of LDH-A by the siRNA. In addition, as far, there is no any single molecule reported that can fully account for Taxol resistance in breast cancer cells. Our results and previous studies suggest that multiple mechanisms may contribute to Taxol resistance and Taxol resistance may be a sum effect of multiple mechanisms/pathways, which suggests that a strategy of combinational therapy is needed to overcome the resistance to Taxol. To identify the molecules that may contribute to Taxol resistance is important for the management of Taxol resistant breast cancer. Nevertheless, our study has shown that the combination of Taxol and LDH-A inhibitor oxamate dramatically increased the inhibitory effect on the growth of Taxol-resistant cancer cells. This potentially can be an effective strategy to overcome Taxol resistance.

The combination of Taxol with oxamate was found to be more effective in killing Taxol-resistant cells, compared to either Taxol or oxamate treatment alone. The combination therapy reveals a synergistic inhibitory effect by promoting breast cancer cell apoptosis (Fig. 6). Apoptosis is a predominant mechanism by which cancer chemotherapeutic agents kill cells [28]. Although oxamate is capable of inhibiting cell cycle progression from G2 to M phase [29], we report here a novel function via inducing apoptotic cell death, with important implications in the clinical treatment of Taxol-resistant cancers, such as breast cancer.

The origin of MDA-MB-435 cells has recently been called into question [30,31]. However, a latest literature indicated that current stocks of both MDA-MB-435 cells and M14 melanoma cells are in fact MDA-MB-435 breast cancer cells instead of M14 melanoma cell line [32]. Nevertheless, we also examined three more breast cancer cell lines, ErbB2-overexpressing BT474 and ErbB2-low-expressing MDA-231 and MCF-7, in order to confirm our findings from MDA-MB-435 cells.

In summary, the present study reveals that LDH-A plays an important role in Taxol-resistance, with Taxol-induced expression and activity of LDH-A serving as an important mechanism for the acquired resistance of human breast cancer cells to Taxol. This study provides valuable information for the development of targeted therapies capable of inhibiting key targets, such as LDH-A. Further studies are needed to demonstrate whether the downregulation of LDH-A mediated re-sensitization of breast cancer cells to Taxol is indeed a consequence of inhibition of glycolysis. Another question arises as to whether the downregulation of other key molecules in the glycolytic pathway may have the same effect as the downregulation of LDH-A. In conclusion, the results of our study highlight the importance of LDH-A in its role in Taxol-resistance and open the door for possible therapeutic interventions in patients that have developed a resistance to Taxol.

## Conclusion

LDH-A plays an important role in Taxol resistance and inhibition of LDH-A re-sensitizes Taxol-resistant cells to Taxol. This study provides valuable information for the future development and use of targeted therapies, such as oxamate, for the treatment of patients with Taxol-resistant breast cancer.

## List of abbreviations

LDH-A: Lactate dehydrogenase-A; DMEM: Dulbecco's modified Eagle medium; c-PARP: the cleaved Poly (ADP-ribose) polymerase; CHX: Cycloheximide.

## Competing interests

The authors declare that they have no competing interests.

## Authors' contributions

MZ designed and carried out the majority of the experiments and drafted the manuscript. YZ, YD, HL, ZL involved in experimental design and carried out some experiments, and helped to revise the manuscript. SK and JL contributed the key reagents. OF, AR, LO, SL helped to revise the manuscript. MT conceived the study and supervised the overall experimental design, execution and revised the manuscript. All authors read and approved the final manuscript.

## Supplementary Material

Additional file 1 **Figure S1.** Knockdown of LDH-A re-sensitizes 435TR1 cell to Taxol by direct cell counting. 435TR1 cells were transfected with scramble siRNA (Ctr) or si-LDHA. 24 hrs after siRNA transfection, cells were treated with 50 nM or 100 nM Taxol for 48 hrs. Cell numbers were directly counted by Typan Blue Staining. Data are presented as the percentage of viability inhibition counted in cells treated without Taxol. *Columns*, mean of three independent experiments; *bars*, SE.*, *P *< 0.05, **, *P *< 0.01. si-LDHA transfection efficiency was showed on the right panel. Click here for file

Additional file 2 **Figure S2. Taxol-resistant cells are more resistant to mitochondrial oxidative phosphorylation inhibitor oligomycin**. MDA-435 and 435TR1 cells were seeded into 96-well plate at density of 5 × 10^3 ^cells per well. 12 hrs after incubation; cells were treated with various concentrations of oligomycin for 24 hrs. Then the cell viability was detected using a MTS reagent, and data are presented as the percentage of viability inhibition measured in cells treated without oligomycin. *Columns*, mean of three independent experiments; *bars*, SE. ***, *P *< 0.001. Click here for file

Additional file 3 **Figure S3.** Combination of Taxol with oxamate shows synergistic inhibitory effects in Taxol-resistant cells by direct cell counting. 435TR1 and 435TRP cells were seeded in 24-well plates and treated with Tax, Oxa alone or Tax plus Oxa with the indicated concentrations for 48 hrs. Cell numbers were counted by Typan Blue Staining. Data are presented as the percentage of viability inhibition counted in cells treated without Tax and Oxa. *Columns*, mean of three independent experiments; *bars*, SE. *, *P *< 0.05. **, *P *< 0.01. ***, *P *< 0.001. Click here for file

Additional file 4 **Figure S4.** Combination of Taxol with oxamate shows better inhibition of MCF7 cells. A, 1 × 10^4 ^per well of MCF7 cells were plated into 96-well plate and treated with Taxol, Oxa, or Tax plus Oxa with indicated concentrations for 48 hrs. Cell viability was examined by MTS assay. Data are presented as the percentage of viability inhibition measured in cells treated without Tax and Oxa. *Columns*, mean of three independent experiments; *bars*, SE.*, *P *< 0.05, **, *P *< 0.01. B, MCF7 cells were treated with 10 nM Taxol or/and 16 mM oxamate for 48 hrs and cell lysates were prepared for Western blotting using antibodies against total PARP (Top) or its cleaved protein c-PARP (Middle). β-actin was used as a loading control (Bottom). Click here for file

Additional file 5 **Figure S5.** The expression of Bcl-2, Bcl-XL, Cdc2 and phosphorylation status of Cdc2 at Tyrosine 15. MDA-435, 435TR1 and TRP cells were collected, lysed and immunoblot analyses were carried out with antibodies against Bcl-2, Bcl-XL, Cdc2 and p-Cdc2-Y15 and tubulin. Click here for file
