# Supplementary material for: Warburg effect in chemosensitivity: Targeting lactate dehydrogenase-A re-sensitizes Taxol-resistant cancer cells to Taxol
Source: Mol Cancer. 2010 Feb 9;9:33. doi: 10.1186/1476-4598-9-33 (PMC2829492; doi:10.1186/1476-4598-9-33)

Supplementary Figure S1 Knockdown of LDH-A re-sensitizes 435TR1 cell to Taxol measured by direct cell counting

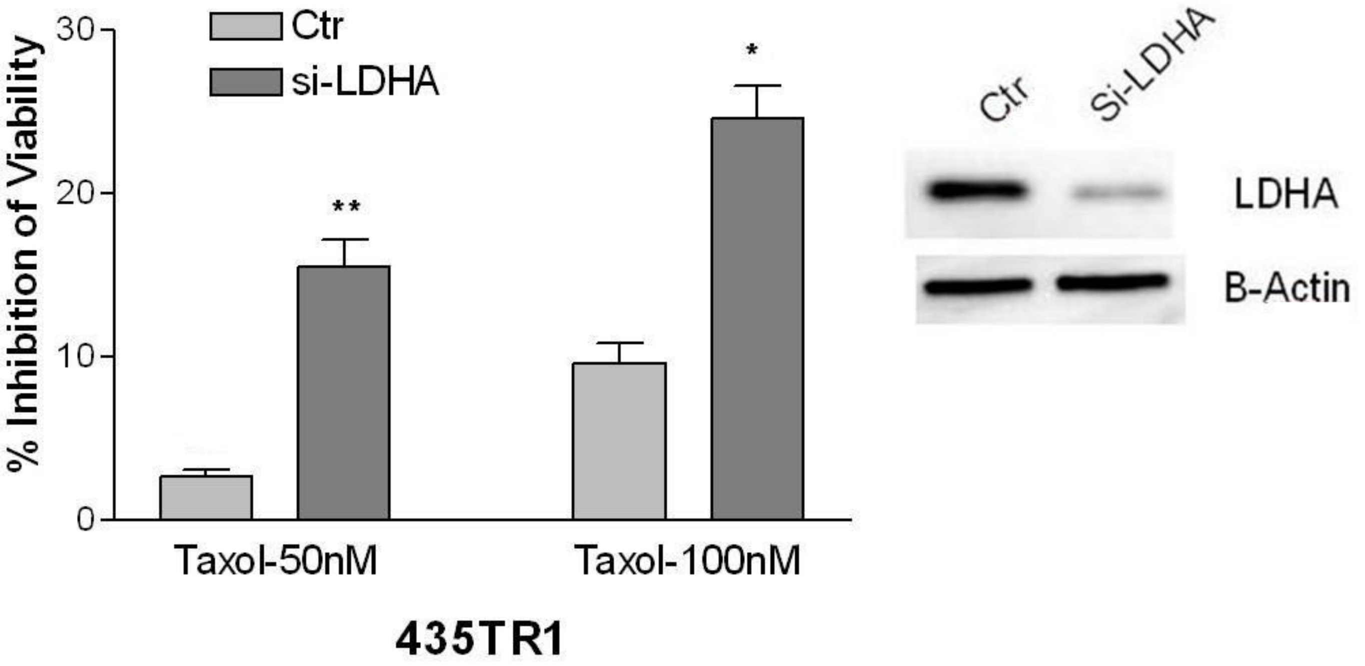

Supplement: Additional file 1 — Figure S1. Knockdown of LDH-A re-sensitizes 435TR1 cell to Taxol by direct cell counting. 435TR1 cells were transfected with scramble siRNA (Ctr) or si-LDHA. 24 hrs after siRNA transfection, cells were treated with 50 nM or 100 nM Taxol for 48 hrs. Cell numbers were directly counted by Typan Blue Staining. Data are presented as the percentage of viability inhibition counted in cells treated without Taxol. Columns, mean of three independent experiments; bars, SE.*, P < 0.05, **, P < 0.01. si-LDHA transfection efficiency was showed on the right panel. [file 1476-4598-9-33-S1.PDF]
