# Supplementary material for: Warburg effect in chemosensitivity: Targeting lactate dehydrogenase-A re-sensitizes Taxol-resistant cancer cells to Taxol
Source: Mol Cancer. 2010 Feb 9;9:33. doi: 10.1186/1476-4598-9-33 (PMC2829492; doi:10.1186/1476-4598-9-33)

Supplementary Figure S2 Taxol-resistant cells are more resistant to mitochondrial inhibitor oligomycin

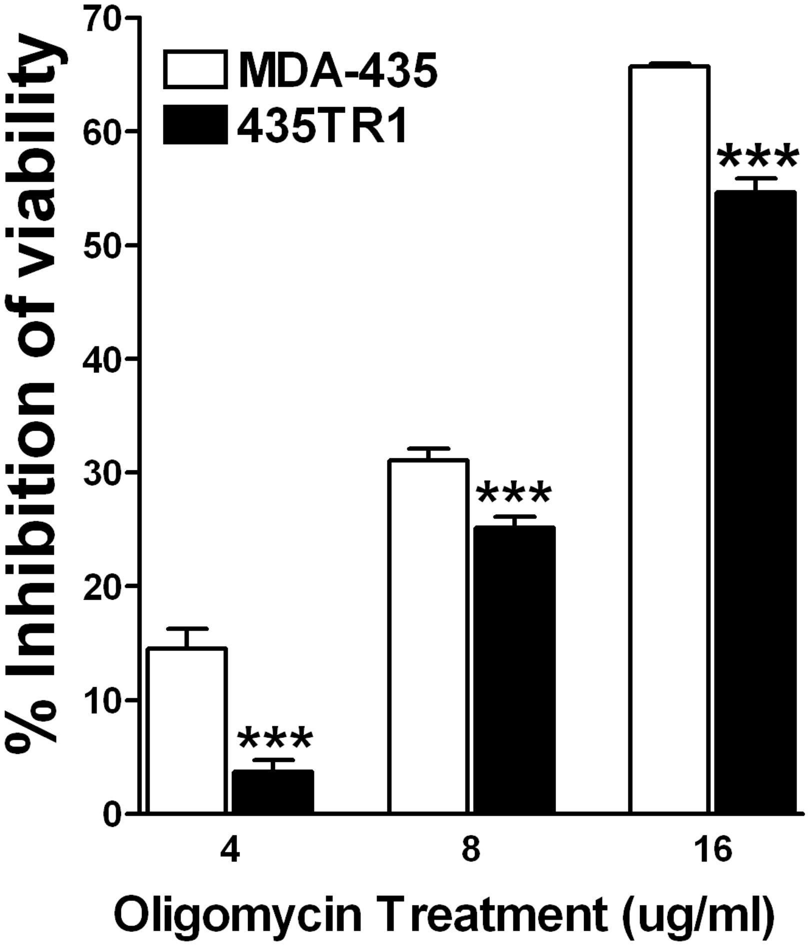

Supplement: Additional file 2 — Figure S2. Taxol-resistant cells are more resistant to mitochondrial oxidative phosphorylation inhibitor oligomycin. MDA-435 and 435TR1 cells were seeded into 96-well plate at density of 5 × 10^3^ cells per well. 12 hrs after incubation; cells were treated with various concentrations of oligomycin for 24 hrs. Then the cell viability was detected using a MTS reagent, and data are presented as the percentage of viability inhibition measured in cells treated without oligomycin. Columns, mean of three independent experiments; bars, SE. ***, P < 0.001. [file 1476-4598-9-33-S2.PDF]
