# Supplementary material for: Warburg effect in chemosensitivity: Targeting lactate dehydrogenase-A re-sensitizes Taxol-resistant cancer cells to Taxol
Source: Mol Cancer. 2010 Feb 9;9:33. doi: 10.1186/1476-4598-9-33 (PMC2829492; doi:10.1186/1476-4598-9-33)

Supplementary Figure S3 Combination of Taxol with oxamate shows synergistic inhibitory effects in Taxol resistant cells by direct cell counting

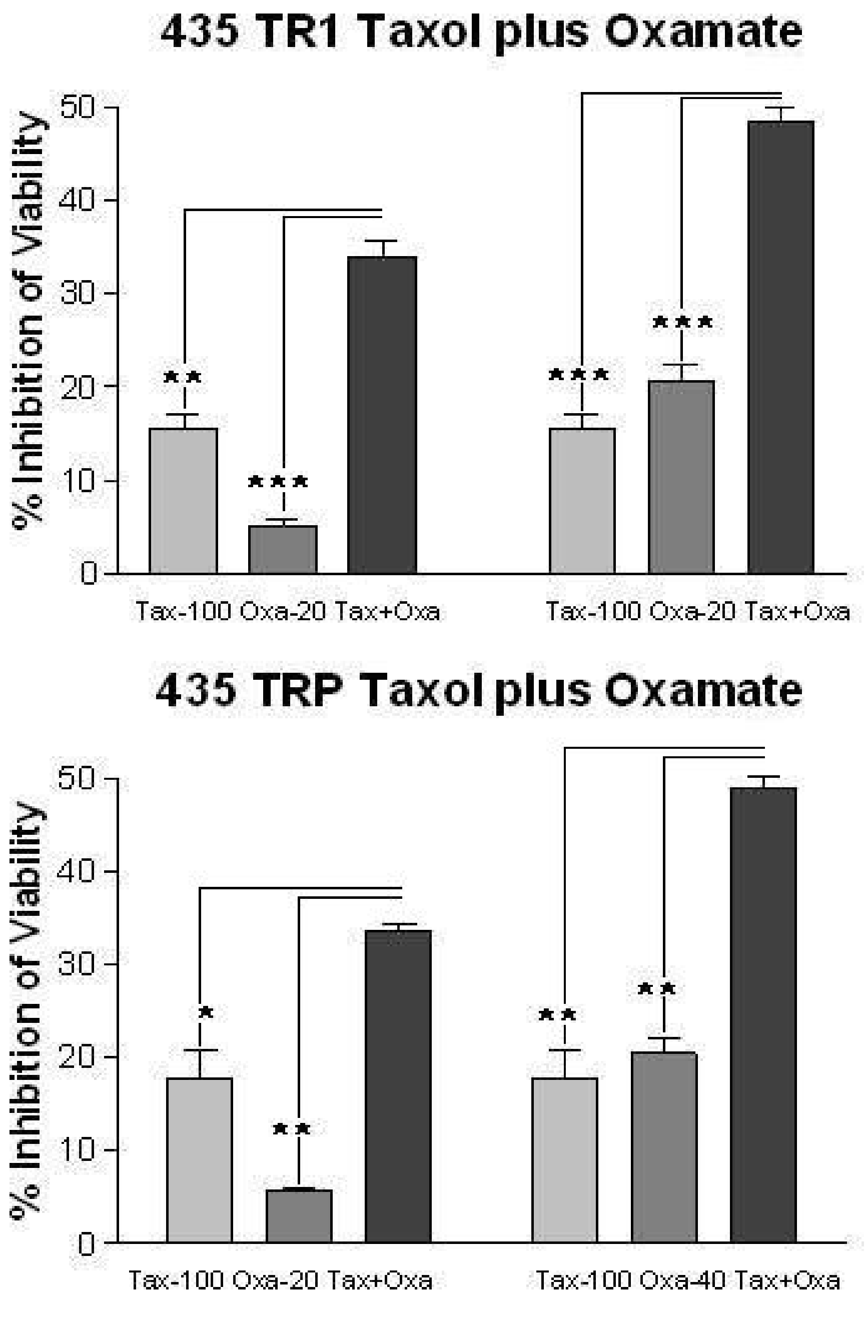

Supplement: Additional file 3 — Figure S3. Combination of Taxol with oxamate shows synergistic inhibitory effects in Taxol-resistant cells by direct cell counting. 435TR1 and 435TRP cells were seeded in 24-well plates and treated with Tax, Oxa alone or Tax plus Oxa with the indicated concentrations for 48 hrs. Cell numbers were counted by Typan Blue Staining. Data are presented as the percentage of viability inhibition counted in cells treated without Tax and Oxa. Columns, mean of three independent experiments; bars, SE. *, P < 0.05. **, P < 0.01. ***, P < 0.001. [file 1476-4598-9-33-S3.PDF]
