# Supplementary material for: Warburg effect in chemosensitivity: Targeting lactate dehydrogenase-A re-sensitizes Taxol-resistant cancer cells to Taxol
Source: Mol Cancer. 2010 Feb 9;9:33. doi: 10.1186/1476-4598-9-33 (PMC2829492; doi:10.1186/1476-4598-9-33)

Supplementary Figure S4 Taxol in combination with oxamate treatment shows better inhibition of MCF7 cells

A

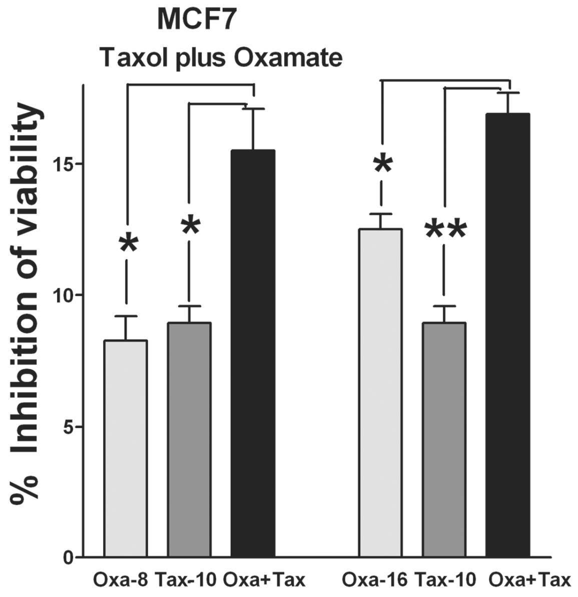

B

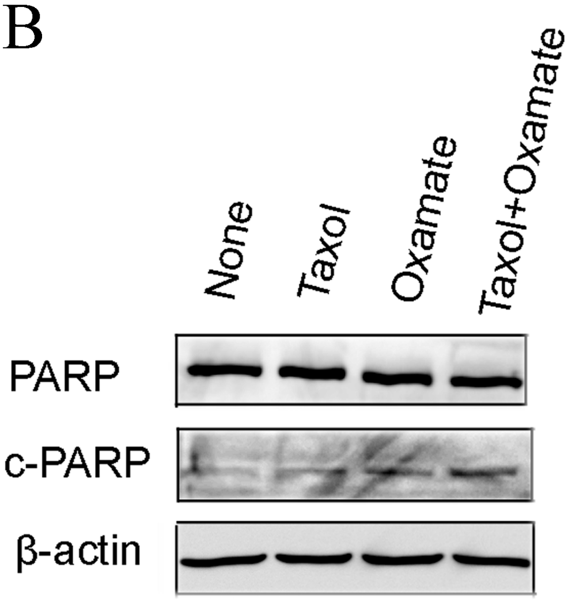

Supplement: Additional file 4 — Figure S4. Combination of Taxol with oxamate shows better inhibition of MCF7 cells. A, 1 × 10^4^ per well of MCF7 cells were plated into 96-well plate and treated with Taxol, Oxa, or Tax plus Oxa with indicated concentrations for 48 hrs. Cell viability was examined by MTS assay. Data are presented as the percentage of viability inhibition measured in cells treated without Tax and Oxa. Columns, mean of three independent experiments; bars, SE.*, P < 0.05, **, P < 0.01. B, MCF7 cells were treated with 10 nM Taxol or/and 16 mM oxamate for 48 hrs and cell lysates were prepared for Western blotting using antibodies against total PARP (Top) or its cleaved protein c-PARP (Middle). β-actin was used as a loading control (Bottom). [file 1476-4598-9-33-S4.PDF]
