# Supplementary material for: Warburg effect in chemosensitivity: Targeting lactate dehydrogenase-A re-sensitizes Taxol-resistant cancer cells to Taxol
Source: Mol Cancer. 2010 Feb 9;9:33. doi: 10.1186/1476-4598-9-33 (PMC2829492; doi:10.1186/1476-4598-9-33)

Supplementary Figure S5 The expression of Bcl-2, Bcl-XL, Cdc2 and phosphorylation statues of Cdc2 at Tyrosine 15

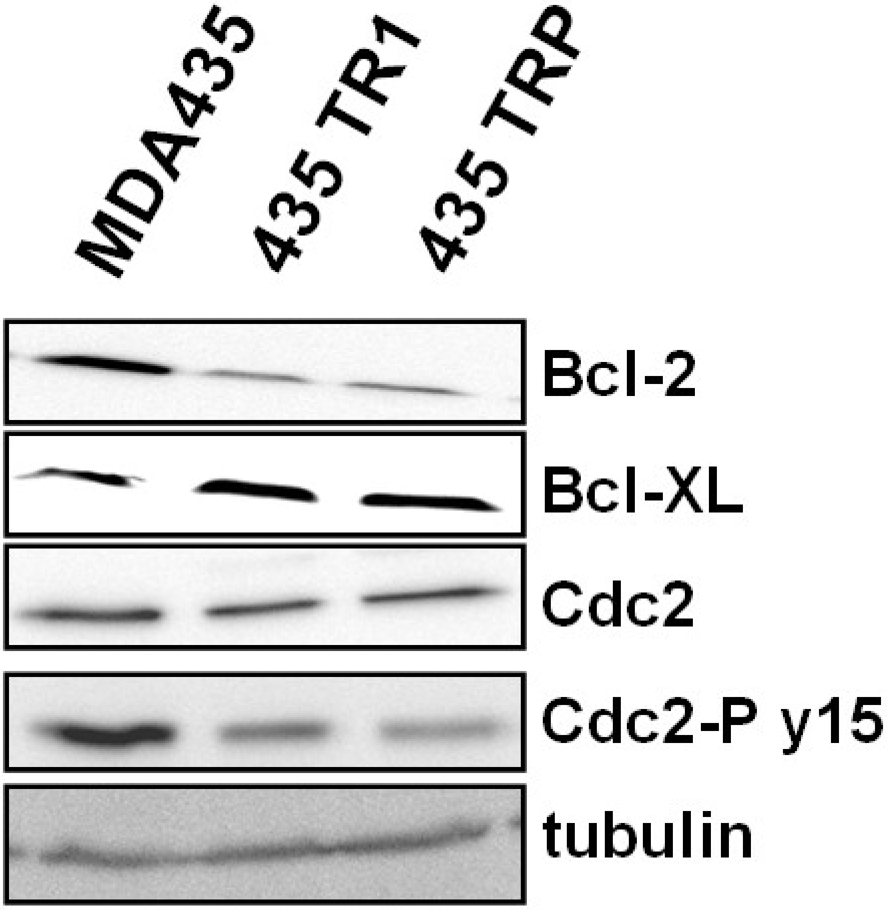

Supplement: Additional file 5 — Figure S5. The expression of Bcl-2, Bcl-XL, Cdc2 and phosphorylation status of Cdc2 at Tyrosine 15. MDA-435, 435TR1 and TRP cells were collected, lysed and immunoblot analyses were carried out with antibodies against Bcl-2, Bcl-XL, Cdc2 and p-Cdc2-Y15 and tubulin. [file 1476-4598-9-33-S5.PDF]
